# Infection in people with heart failure: an overlooked cause of adverse outcomes

**DOI:** 10.1016/j.clinme.2025.100497

**Published:** 2025-08-10

**Authors:** Victoria Palin, Oliver Brown, Fergus Hamilton, Patrick Lillie, Mark Kearney, Richard Cubbon, Michael Drozd

**Affiliations:** a Leeds Institute of Cardiovascular and Metabolic Medicine, School of Medicine, University of Leeds, Leeds, UK; b Infection Sciences, North Bristol NHS Trust, Bristol, UK; c Department of Infection, Castle Hill Hospital, Hull University Hospitals NHS Trust, Kingston Upon Hull, UK; d NIHR Leeds Biomedical Research Centre, Leeds Teaching Hospitals NHS Trust, Chapel Allerton Hospital, Leeds, UK

## Abstract

Infections are a major cause of morbidity and mortality in people with heart failure, accounting for approximately 25% of hospitalisations and deaths. Infection hospitalisations in people with heart failure last twice as long as other hospitalisations, with mortality rates after discharge being comparable to those seen after acute decompensated heart failure. Addressing this major challenge is essential to further improving the survival and quality of life of this population. However, very few studies have sought to understand why people with heart failure are predisposed to adverse infection outcomes and there are currently very few interventions that target this problem. In this review, we explore the underlying factors that may predispose individuals with heart failure to infection, highlight the impact of infections on outcomes, explore the potential strategies that may reduce adverse infection outcomes, and highlight future research priorities.

## Highlights

• Infection is a leading cause of hospitalisation and death in people with heart failure.
• Multimorbidity, frailty, chronic inflammation and suboptimal vaccine response may contribute to increased infection incidence and worse outcomes following an infection event.
• Clinicians should maintain a high index of suspicion for infection, even in the absence of typical signs like tachycardia and pyrexia.
• Management should prioritise infection prevention through vaccination, early detection and timely treatment.
• Further research is needed to better understand the underlying mechanisms and to improve infection prevention and management strategies in populations with heart failure.

## Graphical abstract

**Figure:**
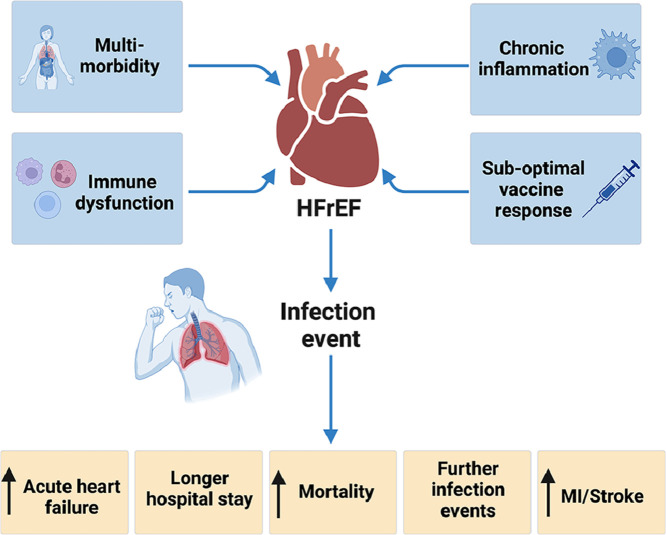

## Introduction

Heart failure is a growing health concern and affects around 900,000 people in the UK. This rise is due to a variety of factors, including advancements in life-prolonging medical therapies for heart failure, advances in the management of ischaemic heart disease, and an ageing population.^1^ There has been a reduction in sudden cardiac death, which is a result of medical therapy and implantable cardioverter-defibrillators.^2^ This shift has effectively unmasked the significant role of non-cardiovascular mortality, with infection accounting for a quarter of all deaths in the heart failure population.^3^ Those with heart failure who are hospitalised due to infection experience adverse short- and long-term outcomes.^4^^,^^5^ This review explores the reasons why people with heart failure are predisposed to infection and outlines potential strategies to prevent and manage this deadly combination.

## Predisposition to infection

Cardiovascular disease increases infection-related mortality,^6^^,^^7^ with heart failure carrying the highest risk among cardiovascular conditions.^8^ This raises the intriguing question of whether the disease process itself predisposes individuals to infection. Key risk factors for fatal infection in people with heart failure and reduced ejection fraction (HFrEF) include the presence of chronical obstructive pulmonary disease (COPD), advancing age, male sex, lower vitamin D levels and higher platelet count.^3^^,^^5^ Pneumonia predominates, accounting for nearly half of infection-related hospitalisations.^4^^,^^5^

While multimorbidity has an important role, patients with heart failure are twice as likely to be admitted with pneumonia compared with age/sex-matched individuals.^9^ Our group has shown that heart failure independently increases infection mortality risk after accounting for other comorbidities.^8^ Post hoc analysis of the PARADIGM-HF and PARAGON-HF trials found that pneumonia incidence in heart failure was three times the expected rate,^10^ suggesting that the disease process itself predisposes to infection beyond frailty alone. However, to fully understand the independent contribution of heart failure will require studies using age- and disease-matched controls.

The pathophysiology of predisposition to infection is not fully understood. The immune system in patients with heart failure might be impaired through several mechanisms related to the systemic inflammatory state.^11^ Trials have consistently shown that these patients have elevated levels of C-reactive protein (CRP) and other inflammatory cytokines.^12^^,^^13^ Chronic systemic inflammation per se identifies people at increased risk of infection death.^14^ This presents an apparent paradox: while systemic inflammation typically reflects upregulation of innate immune components that should theoretically enhance pathogen clearance, these patients experience increased infection susceptibility. This contradiction might be explained by the concept of inflammatory exhaustion or trained tolerance.^15^^,^^16^ Additionally, the COVID-19 pandemic has demonstrated how a pathogen (SARS-CoV-2) can trigger an excessive immune response, leading to a hyperinflammatory state that is severely detrimental.^17^ The relationship is further complicated by the fact it is bi-directional, with heart failure being both a cause and consequence of chronic inflammation.^11^ Indeed, the cytokines tumour necrosis factor (TNF)-α and interleukin (IL)-1 have been shown to impair systolic function.^18^^,^^19^

The issue is complex, as heart failure rarely occurs without comorbidity.^6^ Common comorbidities such as COPD, renal dysfunction and diabetes can further compromise the immune system and increase the risk of infections.^6^ Furthermore, studies have shown suboptimal responses to vaccinations against influenza and herpes zoster virus in people with heart failure.^20^^,^^21^ This impaired adaptability of the immune system in patients with heart failure may contribute to their increased vulnerability to infections and the difficulty in effectively preventing and treating infectious diseases.

Cardiogenic pulmonary oedema may predispose patients with heart failure to respiratory infections, though exact mechanisms remain unclear. Fluid accumulation may compromise local immune defences by impairing mucociliary clearance and hindering immune cell migration, although direct evidence in this context is limited.^22^^,^^23^ This represents an important knowledge gap in understanding the infection susceptibility of patients with heart failure.

Patients with pneumonia exhibit worse heart failure symptoms and poorer quality of life scores in both HFrEF and HFpEF populations. In HFpEF, this is linked to more prior hospitalisation and worse NYHA (New York Heart Association) class, suggesting greater fluid overload.^10^ In HFrEF, higher diuretic doses likely reflect more advanced disease.^5^ Infection is a well-recognised trigger for acute heart failure, increasing myocardial demand and activating neurohormonal pathways,^4^ though whether this relationship is bi-directional remains unclear.

Iatrogenic infection risk in heart failure is low. SGLT2 inhibitors may cause mild, treatable urogenital infections^24^ and cardiac device-related infections are relatively uncommon (<1%), contributing minimally to the overall burden.^25^

## Hospitalisation due to infection

Non-cardiovascular hospitalisations are increasing in heart failure,^26^ with infections accounting for 25% in HFrEF.^5^ Pneumonia is the most common cause (approximately 50%), followed by urinary tract and soft tissue infections.^4^^,^^5^ Infection-related admissions are twice as long and carry considerable higher in-hospital mortality compared to non-infection related admissions.^3^, ^4^, ^5^ In-hospital mortality is the highest for those admitted with pneumonia.^4^ These trends predate the COVID-19 pandemic; therefore it is unsurprising that those with heart failure and COVID-19 infection have been shown to be at increased risk of mechanical ventilation and mortality.^27^

Patients with HFrEF and infection often present atypically compared to those admitted for non-infectious reasons.^5^ Tachycardia and pyrexia, which are common signs of infection, are less frequently observed in patients with heart failure and infection.^5^ The absence of tachycardia may be due to the use of beta blockers for treatment of HFrEF. This atypical presentation can make the early recognition and diagnosis of infection more challenging. Additionally, overlapping symptoms between pneumonia and decompensated heart failure further complicate clinical assessment.

Guideline-directed medical therapy (GDMT) is more often reduced or stopped during infection-related hospitalisation than other for other causes.^28^ This is associated with worse long-term survival.^28^ While adjustments may be necessary due to infection-related complications, failure to promptly reinstate therapy can harm long-term cardiovascular outcomes.

During infection, poor outcomes often stem from immune dysregulation, where the host response causes systemic inflammation and organ dysfunction.^29^ This harmful hyperinflammatory state was evident during COVID-19.^30^ Patients with heart failure likely have impaired immune responses with persistent chronic inflammation,^11^ leading to immune suppressor cell activation.^31^ Combined with age-related immune decline and increased systemic inflammation,^32^ the increased infection mortality may reflect maladaptive immune responses in this ageing heart failure cohort.^30^

## Outcomes following infection

Following infection hospitalisation, people with heart failure have poor short- and long-term outcomes.^4^^,^^5^ An infection-related hospitalisation increases the risk of mortality during follow-up by approximately 1.5 times compared to those admitted without infection.^4^^,^^5^ Alarmingly, the prognosis following an infection hospitalisation is similar to that of a hospitalisation with decompensated heart failure, which is a well-established high-risk event in people with HFrEF.^5^ This risk is even more pronounced when specifically considering pneumonia, with a fourfold increased risk of death from any cause in this population.^10^ Furthermore, for patients with heart failure in the last year of life, most hospitalisations were due to infection.^33^

The elevated risk of mortality is evident within the first 3 months following infection; however, the effect persists, with this population having an increased risk of death over long-term follow-up periods.^4^^,^^10^ This signifies that infection is a major adverse event in a patient’s lifecourse. Furthermore, this poorer survival is not just due to infection or decompensated HF; this population is at increased risk of major cardiovascular events, including myocardial infarction and stroke, after an infection-related hospitalisation.^4^

Following an infection-related hospitalisation, patients with heart failure are significantly more likely to experience a subsequent hospitalisation due to infection compared to any other cause.^5^ This finding suggests the existence of a distinct sub-group of patients who are particularly susceptible to recurrent infection events; however, the underlying pathophysiology of this predisposition is not clear.

## The role of frailty and multimorbidity

As heart failure prevalence and survival increase, the patient population is increasingly older, frail and multimorbid. This poses unique challenges for healthcare as frailty and multimorbidity can present challenges to treatment, adherence to GDMT, survival and quality of life.^34^ Frailty, reflecting reduced physiological reserve, is linked to higher rates of infection-related hospitalisation and worse outcomes.^35^ After admission for pneumonia or heart failure, frail patients experience approximately threefold increased risk of rehospitalisation or mortality within 30 days.^36^ Frailty also limits tolerance to GDMT, with older patients more likely to have ACE-inhibitor dose reductions during admission, which is associated with worse survival.^28^

**Fig. 1:**
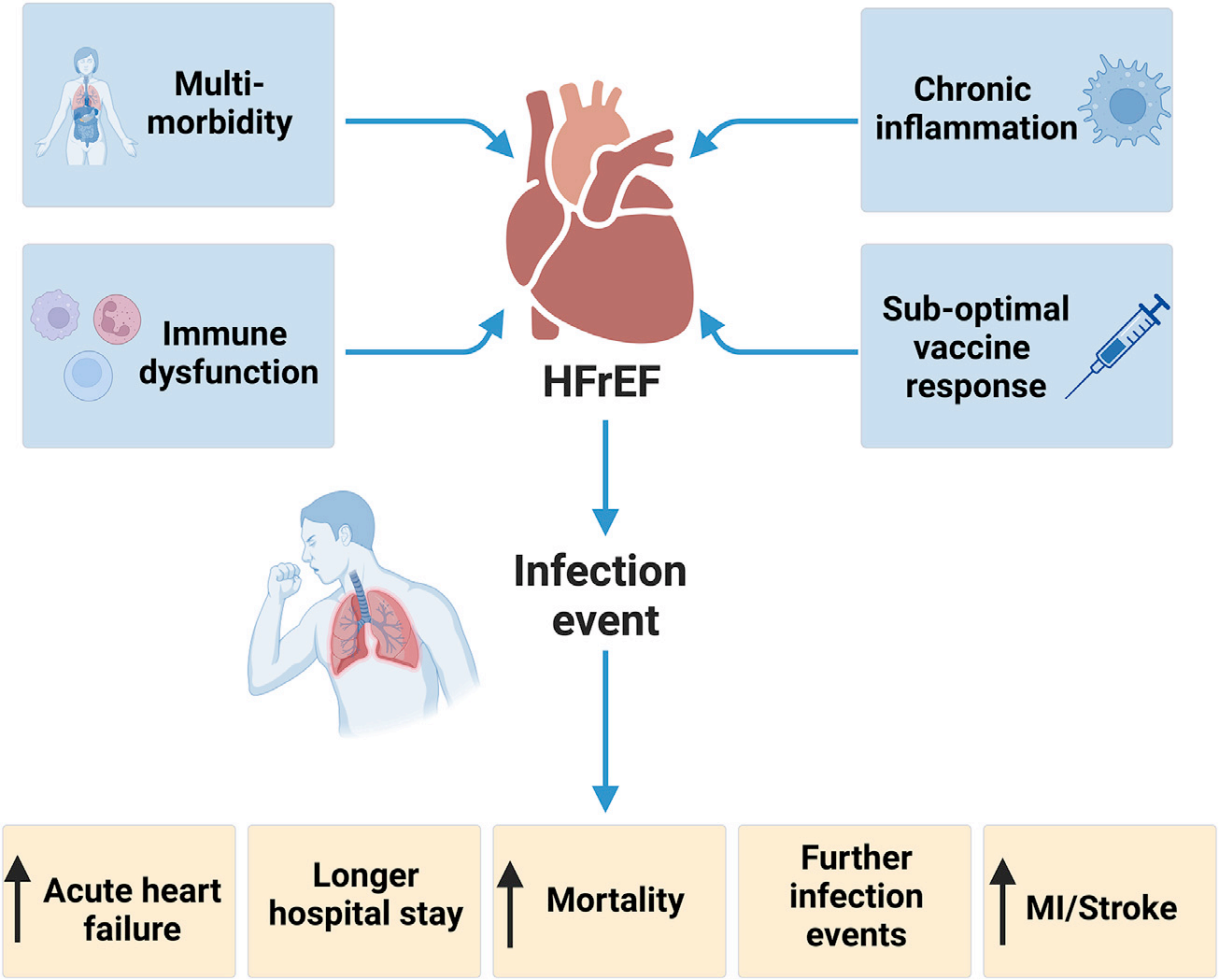
Diagram showing the predisposing factors (blue) that increase the risk of infection events in people with heart failure and reduced ejection. An infection event is associated with adverse outcomes (yellow). Figure created with BioRender.

Frailty and multimorbidity frequently co-exist.^37^ As cardiovascular multimorbidity increases, so does infection-related mortality.^8^ This relationship is observed in both HFrEF and HFpEF populations, particularly among older patients or those with COPD, diabetes or AF, who are more likely to develop pneumonia.^10^ Moreover, multimorbidity also has an additive effect, with the loss of expected life years increasing for each additional condition that a person with heart failure has.^38^ Managing this frail, multimorbid heart failure population requires optimising care beyond standard GDMT to improve outcomes and quality of life (Fig. 1).

## Strategies for reducing infection-related morbidity and mortality in heart failure

While further research is needed to fully understand the complex interplay between heart failure, inflammation and infection, healthcare providers can take proactive steps to reduce the risk of infection-related complications. A comprehensive approach should prioritise infection prevention through vaccination, early recognition and prompt treatment of infections, and tailored care for frail and multimorbid individuals. In the future, more targeted and personalised approaches to managing this vulnerable population may emerge.

Prevention of infection in the population with heart failure is essential in reducing hospitalisation and mortality. The yearly influenza vaccine is associated with reduced all-cause mortality in this population, and is recommended alongside the one-off pneumococcal vaccination by NICE.^39^ The COVID-19 vaccination is also associated with reduced hospitalisations from pneumonia and heart failure.^40^ Targeting the subset of the population with heart failure who are particularly susceptible to infection (previous infection, frail, multimorbid, chronic systemic inflammation) and strongly encouraging vaccination could help reduce morbidity in this population. Although already recommended by most guidelines, vaccination uptake in at-risk groups remains below national targets.^41^ There is currently insufficient evidence to support differential approaches in relation to vaccine type or schedules for patients with heart failure. Further research is required to study the contribution of respiratory synytial virus infection in this population.

Early recognition and prompt treatment of infections in patient with heart failure are critical in improving outcomes.^42^ Healthcare providers should maintain a high index of suspicion for infection when people with heart failure clinically deteriorate, even in the absence of typical signs and symptoms. Use of procalcitonin or rapid PCR testing as tools to detect underlying respiratory infection when there is clinical uncertainty may be useful. Education of patients and their carers about signs and symptoms of potential infection that should prompt rapid medical attention is crucial. Furthermore, the development of risk assessment tools may help identify patients with heart failure who are at the highest risk of infection and require closer monitoring or more aggressive preventive measures. These tools could be integrated into electronic health record systems to alert healthcare providers when high-risk patients present with signs/symptoms suggestive of infection.

Following recovery after infection-related hospitalisation, re-institution of GDMT is important for long-term prognosis. While we acknowledge that in some cases this may not be appropriate, the recognition of this as an indication of advanced frailty can help identify an at-risk group where other interventions like advanced care planning become even more important.^43^ In some cases, it is appropriate to recognise that this person may be in the last year of their life, and high-quality palliative care should become the priority. As influenza vaccination improves outcomes in heart failure,^39^ there is some evidence suggesting that reducing infections is possible and can benefit survival. However, the extent to which this benefit applies across other infections or extends to those with more advanced frailty remains unclear and requires further investigation.

The potential use of immunomodulatory medications targeting molecules involved in the chronic inflammation contributing to the heart failure syndrome is a complex issue, with mixed results in trials. While these drugs may have a potential therapeutic role for heart failure per se, their immunosuppressant effects can increase the risk of infection. Trials often use CRP as a surrogate marker of inflammation, but elevated CRP also is associated with a higher risk of future infection-related death events.^14^

TNF-inhibitors have been disappointing in heart failure. In clinical trials, etanercept showed no clear benefit and was discontinued early,^44^ while infliximab worsened outcomes and increased serious infections.^45^ The IL-1 receptor antagonist anakinra improved VO_2_-max with no difference in infection rates in HFrEF patients with recent heart failure admission and CRP >2 mg/L.^46^ Canakimumab, a monoclonal antibody targeting IL-1β, reduced cardiovascular events in patients with previous myocardial infarction and a CRP >2 mg/L, but increased infection-related deaths.^47^ Post hoc analysis of the CANTOS trial suggested that IL-1β inhibition may improve heart failure outcomes after myocardial infarction,^48^ although such analyses can only be seen as hypothesis generating, rather than informing clinical practice. These mixed data suggest that tested immunomodulatory therapies may have some potential to address heart failure syndrome, although probably worsen the predisposition to infection events.

These findings highlight the need to balance heart failure outcomes with infection risk. Using CRP as a marker to identify patients suitable for these therapies may inadvertently select those at higher risk of infection-related complications.^49^ Future research should identify safer anti-inflammatory targets and better biomarkers for risk stratifying and guiding therapy.

## Limitations in knowledge

Despite the growing evidence for the important role of infection in hospitalisation and mortality in heart failure, there are still significant gaps in our understanding. Most of the available information pertains to the HFrEF cohort, with much less data available for the HFpEF population that accounts for over half of all cases of heart failure. Just as the underlying disease processes in HFrEF and HFpEF may differ, so might the underlying pathophysiology of predisposition to infections. Further investigation into HFpEF is important, as pneumonia has been found to be more common in this population compared to HFrEF.^10^

The definition of infection can be challenging. We propose that clinical trials and observation studies in heart failure would benefit from the development of standardised criteria for the definition of confirmed and probable infection episodes. These would help to compare studies and facilitate high-quality randomised control trials.

The complex interplay between heart failure, infection and chronic inflammation is not yet fully understood. Infection is a common precipitant of heart failure decompensation, making it difficult to attribute a hospital admission to a single cause. In real-world practice, both infection and decompensated heart failure are often treated simultaneously, and it can be unclear which is the primary precipitant. Many people admitted for infection are discharged on higher doses of diuretics,^28^ and infection-related admissions are more likely to be on higher doses of diuretics at admission.^5^ There is currently no evidence to support the use of aggressive diuresis with infection risk. The relationship between pulmonary oedema and the risk of pneumonia (or peripheral oedema and cellulitis) requires further investigation.

The potential impact of heart failure medications on the immune system and susceptibility to infection is also not well understood. Many patients with HFrEF are on multiple medications, including ACE inhibitors / angiotensin blockers / angiotensin receptor-neprilysin inhibitor, beta blockers and aldosterone antagonists. While these medications have proven benefits in managing HFrEF, their effects on immune function and infection risk have not been extensively studied. It is possible that certain medications or combinations of medications may contribute to the increased susceptibility to infection observed. For example, catecholamines have an important role in innate immune training, therefore blockade of these molecules in the treatment of heart failure could alter the immune response.^50^ Investigating the potential immunomodulatory effects of heart failure medications and their influence on infection risk is an important area for future research.

Most studies focus on infections causing hospitalisation and resultant mortality, but many people with heart failure may experience an infection event without being hospitalised. Primary care practice-managed infection is under-researched in this cohort and represents an important area to target for preventing future morbidity and mortality. Diagnosis in the community without investigations may prove even more challenging, as respiratory symptoms can easily be attributed to either decompensated heart failure or pneumonia when the other may be the cause. Future infection events in this population will be complicated by the emergence of antimicrobial resistance. This already at-risk group may be disproportionately affected by these infections worsening, which requires attention.

## Conclusion

In conclusion, infection is an increasingly common cause of hospitalisation and mortality in the population with heart failure, with the complex interplay between heart failure, chronic inflammation and immune dysfunction contributing to their increased susceptibility. Healthcare providers are now dealing with a frailer and multimorbid population with heart failure who are less tolerant to GDMT and more susceptible to infection, necessitating optimisation of their care beyond the four pillars of heart failure treatment. Further research is required to investigate this growing problem, particularly in the HFpEF population and regarding community infection events, and to identify targeted therapies that can effectively modulate the inflammatory response without compromising the immune system’s ability to fight infections. Addressing this complex issue through a comprehensive, multidisciplinary approach that prioritises infection prevention, early recognition and treatment, and personalised care for frail and multimorbid individuals is crucial to reduce infection-related morbidity and mortality in the population with heart failure.

## CRediT authorship contribution statement

**Victoria Palin:** Writing – review & editing, Writing – original draft, Visualization, Methodology. **Oliver Brown:** Writing – review & editing. **Fergus Hamilton:** Writing – review & editing. **Patrick Lillie:** Writing – review & editing. **Mark Kearney:** Writing – review & editing. **Richard Cubbon:** Writing – review & editing, Writing – original draft. **Michael Drozd:** Writing – review & editing, Writing – original draft, Supervision, Methodology, Conceptualization.

## Declaration of competing interest

The authors declare that they have no known competing financial interests or personal relationships that could have appeared to influence the work reported in this paper.
